# Validating a Revised Oral Frailty 5-Item Checklist (OF-5) to Detect Pre-Symptomatic Brain Changes in Cognitively Unimpaired Older Adults

**DOI:** 10.3390/nu17193058

**Published:** 2025-09-25

**Authors:** Makoto Murahashi, Kenjiro Ono, Moeko Noguchi-Shinohara, Mai Ishimiya-Jokaji, Kentaro Ide, Toshihiro Kawano, Shusuke Tokuchi, Risako Suzuki, Mikana Isa, Shuichi Kawashiri, Hiroyuki Nakamura

**Affiliations:** 1 Department of Oral and Maxillofacial Surgery, Graduate School of Medicine, University of the Ryukyus, Okinawa 903-0213, Japan; 2 Department of Neurology and Neurobiology of Aging, Kanazawa University Graduate School of Medical Sciences, Kanazawa 920-8640, Japan; 3 Department of Oral and Maxillofacial Surgery, Kanazawa University Graduate School of Medical Sciences, Kanazawa 920-8640, Japan

**Keywords:** oral frailty, brain atrophy, cognitive decline, magnetic resonance imaging, tooth loss

## Abstract

Objective: Oral frailty is associated with an increased risk of cognitive decline, yet practical tools for early identification remain limited. The Oral Frailty 5-item Checklist (OF-5), recently standardized in Japan, does not account for severe tooth loss, which is a known risk factor for brain atrophy. We developed a revised version of the OF-5 that includes the criterion of having nine or fewer teeth. This study aimed to validate the revised OF-5 as a screening tool for detecting early brain structural changes related to dementia risk in cognitively unimpaired older adults. Methods: We analyzed 732 cognitively unimpaired participants from a population-based Japanese cohort (baseline 2016–2018). Oral frailty was assessed using both the original OF-5 and the revised OF-5. Brain volumes were measured by MRI and processed with FreeSurfer. Associations between oral frailty status and regional brain volumes were tested using multivariable-adjusted models, with further adjustment for nutrient intake and food consumption. Results: The revised OF-5, which adds severe tooth loss (≥9 teeth) as a criterion, showed greater sensitivity in detecting dementia-related brain changes than the original version. With the original OF-5, oral frailty was associated only with reduced fusiform gyrus volume (1.088% vs. 1.109% of estimated total intracranial volume [eTIV]; *p* < 0.05). In contrast, the revised OF-5 detected broader changes: orally frail participants showed significantly higher white matter hyperintensity (WMH) volume (0.366% vs. 0.302% of eTIV; *p* < 0.05) and smaller volumes in the medial temporal lobe (1.824% vs. 1.856%), pars triangularis (0.401% vs. 0.412%), and fusiform gyrus (1.080% vs. 1.111%)—all *p* < 0.05 (FWE-corrected). These associations persisted after adjusting for nutrient intake and food consumption. Conclusions: The revised OF-5 improves identification of pre-symptomatic brain changes in cognitively healthy older adults, independent of nutrition. It may serve as a simple and practical tool for early screening of dementia risk in clinical and community settings.

## 1. Introduction

In a world where the population is aging rapidly, the number of cases of cognitive decline is increasing, and in Japan, it is predicted that by 2040, approximately 15% of adults aged 65 and over will develop dementia [1]. This trend reflects a global challenge in healthcare, with dementia becoming a leading cause of disability and dependency among older adults worldwide [2]. While research has traditionally focused on direct neurological markers of cognitive decline, emerging evidence suggests a complex relationship between oral health and brain function [3]. Recent studies have shown that oral frailty, characterized by a decline in oral function and health status, may serve as an early indicator of cognitive decline [4]. Our research has recently revealed a significant finding: severe tooth loss (≤9 teeth) is associated with parahippocampal gyrus atrophy and increased white matter hyperintensity (WMH) volume, both characteristics of dementia, even in cognitively normal individuals [5]. This discovery suggests that dental status may serve as an early marker for brain structural changes associated with cognitive decline. Early identification and intervention in these risk factors have shown significant potential in preventing or delaying cognitive decline [6,7].

Despite growing evidence linking oral health to brain structure and cognitive function, several critical knowledge gaps remain unexplored. The oral frailty five-item checklist (OF-5), recently adopted as a standardized assessment tool by three Japanese academic societies in 2024 [8], provides a comprehensive evaluation of oral frailty. Based on our findings regarding the association between tooth loss and brain structural changes characteristic of dementia [5], we have developed a revised version of the OF-5 that incorporates having nine or fewer remaining teeth as an additional criterion. The relationship between oral health and cognitive function involves complex pathways, including nutritional status and inflammatory processes [9]. However, the comparative effectiveness of the standard OF-5 and our proposed revised version in predicting dementia risk through brain structural changes requires thorough investigation.

Oral frailty is now understood as a multidimensional condition that includes both clinical and functional impairments. These range from measurable dental status (e.g., tooth loss) to subjective difficulties such as chewing, swallowing, and speech. Each component may reflect distinct pathways—nutritional, neuromuscular, and neurocognitive—that could contribute to dementia risk. Distinguishing these dimensions may allow for more targeted assessments and interventions.

Based on our prior findings that severe tooth loss (≤9 teeth) was associated with parahippocampal atrophy and increased white matter hyperintensity [5], we hypothesized that adding this objective criterion to the OF-5 checklist would improve its sensitivity in detecting brain structural changes associated with early dementia risk. The cutoff of nine or fewer teeth is supported by Japanese national oral health surveys and has been associated with both nutritional deficits and cognitive decline [5,10]. However, the OF-5 has not yet been validated in populations outside of Japan, and its generalizability to other cultural or healthcare settings remains uncertain.

Therefore, in this study, we prospectively validated a revised OF-5 that includes severe tooth loss as an additional item. Our aim was to determine whether this version more effectively identifies dementia-related brain changes in cognitively unimpaired older adults. We also tested whether these associations remain significant after accounting for dietary intake and nutrition-related confounders. This work may support the development of a more sensitive and clinically useful screening tool for preclinical dementia risk.

## 2. Methods

### 2.1. Study Population

**Figure 1:**
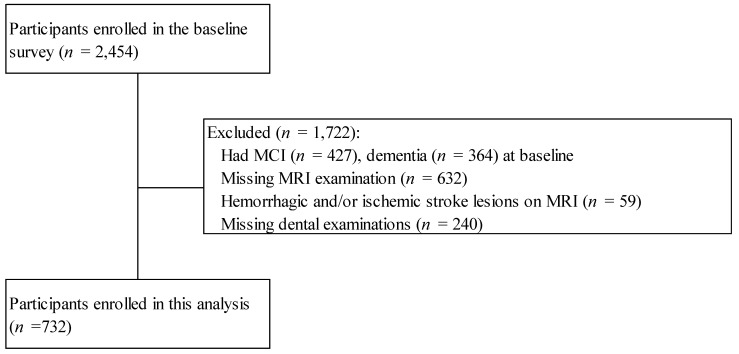
Flow chart of participant enrollment. MCI, mild cognitive impairment; MRI, magnetic resonance imaging.

The baseline survey was conducted in Nakajima, situated in Nanao City within the Ishikawa Prefecture, Japan, between 2016 and 2018 in a population-based, longitudinal cohort of individuals within a specific age range. A detailed description of the baseline survey has been previously described [5,10,11]. Participants were recruited through local health outreach programs, mailed invitations, and community announcements, aiming to include all adults aged ≥60 years. The study included 2454 inhabitants aged ≥60 years, representing 92.9% of the total population within this age group. While randomization was not applied, the near-exhaustive sampling approach minimizes selection bias and enhances representativeness. The participants comprised both community-dwelling older adults and institutionalized individuals. We excluded individuals who had mild cognitive impairment (MCI) (*n* = 427) or dementia (*n* = 364) at the baseline. We also excluded individuals who had no brain magnetic resonance imaging (MRI) examinations (*n* = 632), no dental examinations (*n* = 240), and hemorrhagic and/or ischemic stroke lesions on MRI regardless of the presence or absence of neurological symptoms (*n* = 59). Stroke lesions (hemorrhagic and/or ischemic) were identified by two trained neuroradiologists who were blinded to the clinical information. Finally, data from 732 individuals were included in the analysis (Figure 1). Multivariable statistical models were applied to adjust for potential confounders such as age, sex, education, vascular risk factors, and health behaviors, thereby reducing residual confounding.

Although data collection occurred between 2016 and 2018, the delay in reporting was due to the extended time required for standardized dental verification, MRI data preprocessing using FreeSurfer version 5.3 (http://surfer.nmr.mgh.harvard.edu), and multi-institutional ethical approvals across collaborating centers. Additionally, the formal standardization of the original OF-5 checklist by academic societies in 2024 provided an important benchmark, allowing for a more meaningful validation of our revised version within a recognized framework.

This study complied with the principles outlined in the Declaration of Helsinki, and all method-ologies were approved by the Kanazawa University Medical Ethics Review Committee (Approval Number 933, 1117, 1188, 2186) and the Ryukyu University Medical Ethics Review Committee (Approval Number 2153).

### 2.2. Cognitive Status

“Cognitively unimpaired” status was defined as the absence of MCI or dementia based on structured clinical assessment. Diagnosis of dementia was based on the Diagnostic and Statistical Manual of Mental Disorders, third edition, revised (DSM-III-R) [12], whereas diagnosis of MCI was established according to the International Working Group on general criteria for MCI [13], which stated that (I) persons should be judged as abnormal using other modalities besides not fulfilling the DSM-III-R dementia criteria, (II) functional activities of the person are mainly preserved or at least impairment is minimal, and (III) the person should have evidence of cognitive decline, either by self-assessment and/or the use of an informative report in conjunction with deficits on objective cognitive tasks. Among participants without dementia, a Clinical Dementia Rating score of 0.5 was used as the objective cognitive impairment value to denote cognitive and functional impairment consistent with MCI. A detailed description of the data has been previously published [5].

### 2.3. Assessment of Oral Frailty

This study evaluated oral frailty and its phenotype based on five criteria: remaining-tooth count, chewing ability, swallowing function, oral moisture, and pronunciation clarity. Oral frailty was assessed using the original OF-5 [8,14] and the revised OF-5 classification developed in this study.

A dentist conducted comprehensive dental examinations for all participants, adhering to the Third National Health and Nutrition Examination Survey guidelines [15]. The tooth categories included healthy, carious, and treated teeth (encompassing crowned, inlay, and abutment teeth for prosthetic devices), as well as fully erupted third molars. Excluded were unerupted or congenitally absent teeth, root remnants, and excessively mobile teeth. The study defined “fewer teeth” as 19 or fewer remaining teeth, and “severe tooth loss” as nine or fewer remaining teeth (1 point). Chewing difficulty was assessed using responses to the self-reported question “Do you have difficulty eating hard foods compared to six months ago?” (“Yes” = 1 point). Swallowing difficulty was assessed using responses to the self-reported question “Do you sometimes choke on tea, soup, etc.?” (“Yes” = 1 point). Dry mouth was assessed using responses to the self-reported question “Do you feel dry mouth?” (“Yes” = 1 point). Pronunciation difficulty was assessed using responses to the self-reported question “Do you have trouble pronouncing words clearly in everyday conversation?” (“Yes” = 1 point). Based on these criteria, we classified oral frailty into two groups based on the OF-5 and the revised OF-5 checklists (Table 1).

### 2.4. Magnetic Resonance Imaging Analysis

Structural MRI was conducted using a 1.5-T system (ECHELON RX; Hitachi, Japan). A detailed description of brain MRI data acquisition and processing has been previously published [5]. Three-dimensional volumetric acquisition of T1-weighted turbo field echo images was conducted according to the brain MRI protocol for the Alzheimer’s Disease Neuroimaging Initiative study [16] (Echo time/Repetition time, 4.0/9.2 ms; flip angle, 8°; Field of View, 240 mm; acquisition matrix, 192 × 192; number of slices, 170; voxel size, 0.9375 × 0.9375 mm^2^; slice thickness, 1.2 mm). All T1-structural images were processed using FreeSurfer version 5.3 (http://surfer.nmr.mgh.harvard.edu) [17] at Tohoku University following standard preprocessing pipelines. Quality control (QC) was conducted in two stages: (1) automated detection of outlier segmentations based on total brain volume and individual regional volumes, and (2) visual inspection by trained raters for errors in skull stripping, pial surface reconstruction, and cortical labeling. Scans with segmentation failures, major artifacts, or anatomical distortions were excluded from the analysis. These QC steps ensured the accuracy and reliability of volumetric measures used in the study. Volumes of the areas of interest were created using FreeSurfer [18]. The 37 areas were calculated as the sum of the volumes of the right and left sides of each area. The TBV was calculated by summing the white and gray matter volumes. WMHs were identified based on their characteristic appearance as hypointense regions on T1-weighted MRI, consistent with manifestations of small vessel ischemic disease. Although FLAIR imaging is typically preferred for WMH detection, only T1-weighted sequences were available in this cohort. WMH segmentation was performed using an automated tissue classification approach integrated within FreeSurfer (v5.3). All segmentations were visually inspected for quality control by two trained neuroradiologists blinded to participant clinical data. Scans with major artifacts or segmentation errors were excluded from the analysis. The estimated total intracranial volume (eTIV) was used to normalize the volumetric value.

### 2.5. Nutritional Status

Dietary intake was assessed using a self-administered Food Frequency Questionnaire (FFQ). This questionnaire has been validated and reported [5,19]. Each participant completed the questionnaire in advance, and trained dieticians checked it during the screening test. The average food intake per day was calculated using the frequency of meals per week and the amount of each food portion. The nutritional intake was determined using the Standard Tables of Food Composition in Japan (5th Revised Edition) [20]. The FFQ assessed the intake of major macronutrients (carbohydrates, proteins, fats) and micronutrients (vitamins, minerals) as well as specific food groups, including fruits, vegetables, grains, dairy, meat, fish, and beverages. All dietary nutrients were adjusted for total energy using the density method [21].

### 2.6. Statistical Analysis

To compare participant characteristics, we used one-way analysis of variance for the mean calculations of the continuous variables and chi-square test for the categorical variables. A two-tailed *p*-value < 0.05 indicates statistical significance. Analysis of covariance (ANCOVA) was employed to estimate and compare the multivariable-adjusted values and their 95% confidence intervals for oral frailty. In the multivariable-adjusted analysis, age, sex, educational level, hypertension, diabetes mellitus, LDL and HDL cholesterol levels, BMI, smoking habits, and exercise habits were analyzed as covariates. We applied family-wise error (FWE) correction for multiple comparisons using the Bonferroni method. Brain regions associated with early dementia pathology (e.g., medial temporal lobe, fusiform gyrus, pars triangularis) were considered confirmatory targets based on prior hypotheses. Other brain regions and dietary variables were analyzed in an exploratory manner. FWE-corrected *p*-values < 0.05 were considered statistically significant for confirmatory analyses. For exploratory comparisons, uncorrected *p*-values are reported with interpretation made cautiously. The SPSS software suite (version 26; SPSS Inc., Chicago, IL, USA) was used for all statistical analyses.

## 3. Results

### 3.1. Participant Characteristics According to the Oral Frailty Status

The study included 732 participants, with 394 (53.8%) classified as robust and 338 (46.2%) as orally frail according to the OF-5 criteria. The mean age of the participants with oral frailty (71.75 ± 6.97 years) was significantly higher than that of the robust participants (69.08 ± 5.80 years; *p* < 0.05). Women constituted 56.9% of the total sample, with similar proportions in both groups (robust: 56.1%, oral frail: 58.0%). Serum HDL cholesterol levels were significantly lower in the oral frailty group (59.50 ± 13.80 mg/dL) than in the robust group (62.46 ± 16.08 mg/dL; *p* < 0.05). The prevalence of major health conditions, including hypertension (52.4%) and diabetes mellitus (15.4%), did not differ significantly between the groups (Table 2).

Regarding oral health parameters, the oral frailty group exhibited markedly higher prevalence rates across multiple components. The proportion of participants with 19 or fewer teeth was substantially higher in the oral frailty group (85.2%) than in the robust group (27.7%; *p* < 0.05). Similarly, severe tooth loss (≤9 teeth) affected 53.8% of the oral frailty group versus 14.7% of the robust group (*p* < 0.05). Chewing difficulties were reported by 87.3% of participants with oral frailty compared with 18.8% of robust participants (*p* < 0.05). Swallowing difficulties and dry mouth were also significantly more prevalent in the oral frailty group (46.2% and 21.6%, respectively) than in the robust group (11.2% and 2.3%, respectively; *p* < 0.05) (Table 2).

In the revised OF-5 analysis (*n* = 732), similar patterns emerged with 467 participants classified as robust and 265 as orally frail. The revised classification maintained significant differences in age, HDL cholesterol levels, and all oral health parameters between the groups. Exercise habits emerged as significantly different in the revised OF-5 analysis, with 46.9% of the robust participants reporting regular exercise compared to 39.5% in the oral frailty group (*p* < 0.05) (Table 2).

### 3.2. Association Between Oral Frailty and Regional Brain Volumes in Cognitively Unimpaired Older Adults

Analysis of brain volumes in relation to oral frailty status revealed several significant associations after adjusting for multiple confounding factors including age, sex, educational level, hypertension, diabetes mellitus, BMI, smoking, exercise habits, and cholesterol levels (Table 3). Using the original OF-5 criteria, participants with oral frailty showed significantly smaller fusiform gyrus volume compared with the robust group (1.088% vs. 1.109% of estimated total intracranial volume [eTIV]; *p* < 0.05, FWE-corrected).

The associations between oral frailty and brain volume became more pronounced when using the revised OF-5 classification. Participants classified as orally frail demonstrated significantly higher WMH volume (0.360% vs. 0.305% of eTIV; *p* < 0.05, FWE-corrected) compared with the robust group. Additionally, the orally frail group showed significantly reduced volumes in several brain regions, including the pars triangularis gyrus (0.402% vs. 0.412% of eTIV), medial temporal lobe (1.824% vs. 1.856% of eTIV), and fusiform gyrus (1.080% vs. 1.111% of eTIV; all *p* < 0.05, FWE-corrected). Other examined brain regions, including the total brain volume, frontal lobe, lateral temporal lobe, parietal lobe, occipital lobe, insula, cingulate cortex, hippocampus, and amygdala, showed no significant differences between the oral frailty and robust groups in either classification system.

### 3.3. Nutrient Intake Patterns Associated with the Oral Frailty Status in Cognitively Unimpaired Older Adults

Analysis of dietary intake revealed significant differences in nutrient consumption between the orally frail and robust participants after adjusting for potential confounding factors (Table 4). Using the original OF-5 criteria, participants with oral frailty showed significantly lower intake of several key nutrients compared with the robust group. Total dietary fiber intake was notably lower in the oral frailty group (12.46 g vs. 13.86 g; *p* < 0.001), with reductions in both insoluble (8.57 g vs. 9.47 g; *p* < 0.001) and soluble dietary fiber (2.84 g vs. 3.14 g; *p* < 0.01).

The oral frailty group also demonstrated significantly lower intake of various micronutrients, including iodine (1858.06 μg vs. 2250.91 μg; *p* < 0.001), potassium (2668.51 mg vs. 2886.11 mg; *p* < 0.01), and vitamin K (256.36 μg vs. 286.75 μg; *p* < 0.01). Additionally, participants with oral frailty consumed less folic acid (341.05 μg vs. 373.26 μg; *p* < 0.01) and vitamin C (103.03 mg vs. 113.88 mg; *p* < 0.01) compared with the robust participants.

Similar patterns were observed when using the revised OF-5 classification. Notably, the strongest associations were found for iodine intake (1802.26 μg vs. 2221.82 μg; *p* < 0.001), total dietary fiber (12.35 g vs. 13.70 g; *p* < 0.001), and insoluble dietary fiber (8.53 g vs. 9.36 g; *p* < 0.01). The revised classification also identified significant differences in mineral intake, with the oral frailty group showing lower levels of copper (1.12 mg vs. 1.19 mg; *p* < 0.01) and manganese (3.42 mg vs. 3.70 mg; *p* < 0.01) consumption compared with the robust group.

### 3.4. Food and Beverage Consumption Patterns Associated with the Oral Frailty Status in Cognitively Unimpaired Older Adults

Analysis of food and beverage consumption patterns revealed significant differences between the orally frail and robust participants after adjusting for potential confounding factors (Table 5). Using the original OF-5 criteria, participants with oral frailty showed markedly lower consumption of vegetables compared to the robust group. Specifically, consumption of vegetables not high in beta-carotene (130.47 g vs. 153.08 g; *p* < 0.001) and other vegetables (130.45 g vs. 153.37 g; *p* < 0.001) was reduced in the oral frailty group. Additionally, the oral frailty group consumed significantly less seaweed (12.17 g vs. 14.82 g; *p* < 0.001) and nuts (1.76 g vs. 3.12 g; *p* < 0.001).

Conversely, participants with oral frailty showed higher consumption of certain fats and oils. Lard consumption was significantly higher in the oral frailty group (7.40 g vs. 6.09 g; *p* < 0.01), as was the total fat and oil intake (9.11 g vs. 7.81 g; *p* < 0.01). The oral frailty group also consumed more cider (12.24 g vs. 3.51 g; *p* < 0.01) and udon noodles (14.54 g vs. 9.14 g; *p* < 0.05) compared with the robust group.

Similar patterns emerged when using the revised OF-5 classification. Seaweed consumption remained significantly lower in the oral frailty group (11.81 g vs. 14.61 g; *p* < 0.001), whereas fat and oil consumption was higher (9.42 g vs. 7.84 g; *p* < 0.01). The revised classification also identified significantly lower consumption of rolled oats (0.01 g vs. 0.56 g; *p* < 0.01) and nuts (1.68 g vs. 2.96 g; *p* < 0.01) in the oral frailty group. Additionally, the oral frailty group showed higher consumption of canned coffee (30.90 g vs. 14.42 g; *p* < 0.05) compared with the robust group.

### 3.5. Association Between Oral Frailty and Regional Brain Volumes After Adjustment for Nutrient Intake in Cognitively Unimpaired Older Adults

To examine whether the observed associations between oral frailty and brain volumes were mediated by nutritional factors, we conducted additional analyses adjusting for nutrient intake variables that showed significant associations in our previous analysis (Table 6). Using the original OF-5 criteria, a significant association with fusiform gyrus volume persisted after adjustment for nutrient intake (1.088% vs. 1.109% of estimated total intracranial volume [eTIV]; *p* < 0.05, FWE-corrected).

In the revised OF-5 classification, the associations between oral frailty and brain volumes remained significant after controlling for nutrient intake. Specifically, the WMH volume remained significantly higher in the oral frailty group (0.366% vs. 0.302% of eTIV; *p* < 0.05, FWE-corrected). Additionally, the orally frail group continued to show significantly smaller volumes in the pars triangularis gyrus (0.401% vs. 0.412% of eTIV), medial temporal lobe (1.824% vs. 1.856% of eTIV), and fusiform gyrus (1.080% vs. 1.111% of eTIV; all *p* < 0.05, FWE-corrected). These persistent associations after adjustment for nutrient intake suggest that the relationship between oral frailty and brain volume differences may be independent of nutritional factors.

### 3.6. Association Between Oral Frailty and Regional Brain Volumes After Adjustment for Food and Beverage Intake in Cognitively Unimpaired Older Adults

To investigate whether dietary patterns influenced the relationship between oral frailty and brain volumes, we performed additional analyses adjusting for food and beverage intake variables that showed significant associations in our previous analysis (Table 7). Using the original OF-5 criteria, the significant association with fusiform gyrus volume remained after adjustment for food and beverage intake (1.088% vs. 1.109% of estimated total intracranial volume [eTIV]; *p* < 0.05, FWE-corrected).

In the revised OF-5 classification, several significant associations persisted after controlling for food and beverage intake. WMH volume remained significantly higher in the oral frailty group (0.361% vs. 0.304% of eTIV; *p* < 0.05, FWE-corrected). The orally frail group continued to show significantly smaller volumes in the pars triangularis gyrus (0.401% vs. 0.412% of eTIV), medial temporal lobe (1.827% vs. 1.855% of eTIV), and fusiform gyrus (1.081% vs. 1.110% of eTIV; all *p* < 0.05, FWE-corrected). The persistence of these associations after adjustment for food and beverage intake suggests that the relationship between oral frailty and brain volume differences may be independent of specific dietary patterns.

## 4. Discussion

This study yielded two significant findings regarding the relationship between oral frailty and brain structural changes in cognitively unimpaired older adults. First, we found that individuals classified as orally frail using our revised OF-5 criteria, which includes having nine or fewer remaining teeth, showed significantly higher WMH volume and reduced volumes in specific brain regions, including the pars triangularis gyrus, medial temporal lobe, and fusiform gyrus, compared with the robust group. Second, these associations between oral frailty and brain structural changes remained significant even after adjusting for nutritional factors and dietary patterns, suggesting that the relationship between oral frailty and brain volume differences may be independent of nutritional status. These findings extend our previous research on the association between severe tooth loss and brain structural changes by demonstrating the potential utility of our revised OF-5 criteria in identifying individuals who may be at risk for cognitive decline.

The observed association between oral frailty (as assessed by our revised OF-5) and specific brain structural changes merits detailed examination. The increased WMH volume and reduced volumes in the medial temporal lobe and fusiform gyrus among orally frail individuals align with the established patterns of brain atrophy associated with cognitive decline. The observed differences in regional brain volumes, while small in absolute eTIV percentages, correspond to moderate standardized effect sizes. For example, a 0.055% difference in WMH volume translates to an approximate increase of ~5–6 mL in white matter lesion load. These standardized values enhance interpretability and suggest that the associations are clinically meaningful, even in cognitively unimpaired individuals. The fusiform gyrus has been specifically implicated in the development of dementia, with studies demonstrating that patients with amnestic mild cognitive impairment exhibit degenerative structural changes in this region [22]. Similarly, atrophy in the medial temporal lobe, particularly in structures such as the parahippocampal gyrus, has been linked to impairments in episodic memory formation and is considered a hallmark of early Alzheimer’s disease pathology [23]. The presence of increased WMH volume is particularly noteworthy because it indicates potential cerebrovascular pathology, which has been associated with cognitive decline and dementia risk [5]. Our finding that these brain structural changes are detectable in cognitively unimpaired individuals classified as orally frail using our revised OF-5 criteria suggests that this assessment tool may be valuable for identifying individuals at risk for cognitive decline before clinical symptoms manifest.

The persistence of the associations between oral frailty and brain structural changes after adjusting for nutritional factors and dietary patterns suggests the existence of alternative pathways linking oral health to brain structure. While previous research has emphasized the role of nutritional status as a mediator between oral health and cognitive function [24], our findings indicate that the relationship between oral frailty and brain volume differences may be more complex. This independence from nutritional factors aligns with emerging evidence suggesting multiple potential mechanisms linking oral health to brain structure, including inflammatory processes and vascular pathways [3]. Poor oral health has been associated with systemic inflammation and specific epigenetic changes that can affect brain structure and function independently of nutritional status [25]. In addition to inflammatory and vascular mechanisms, psychosocial and behavioral pathways may also contribute to the observed associations. Oral frailty can lead to reduced dietary enjoyment, social withdrawal, and lower quality of life, all of which have been independently associated with brain atrophy and cognitive decline. Furthermore, oral frailty may indirectly reflect broader functional decline or systemic frailty that includes subclinical neurodegeneration. The revised OF-5, by including severe tooth loss, may better capture individuals at this higher level of cumulative health risk. Unlike biomarkers or advanced imaging, this checklist can be administered quickly in community or dental settings, making it a feasible pre-screening tool for identifying those who may benefit from further neurological evaluation or early intervention. From a public health perspective, implementing the revised OF-5 in routine dental checkups or community screenings could enable earlier identification of at-risk older adults, particularly in aging societies such as Japan. As the aging population increases globally, the importance of accessible and non-invasive screening tools like the revised OF-5 will become more critical. Furthermore, studies have identified differential gene expression patterns in the fusiform gyrus that may explain its vulnerability to structural changes in the context of poor oral health [26]. The robustness of these associations after controlling for nutritional factors not only validates the utility of our revised OF-5 criteria but also suggests that oral frailty assessment could provide unique insights into cognitive decline risk beyond what can be explained by nutritional status alone.

The findings of this study have significant implications for clinical practice and public health strategies in the context of dementia prevention. Because the checklist can be administered easily in community or primary care settings, it may enable earlier intervention for modifiable dementia risk factors. This is particularly relevant in rapidly aging populations such as Japan, where proactive strategies are needed to identify preclinical risk. Early identification of individuals at risk of cognitive decline is crucial because interventions are most effective when implemented before significant brain changes occur [6]. The Lancet Commission on dementia prevention has emphasized the importance of identifying modifiable risk factors, including oral health, in preventing cognitive decline [2]. Our revised OF-5, which incorporates the criterion of having nine or fewer remaining teeth, could serve as a practical and noninvasive screening tool for identifying individuals who may benefit from early intervention. This is particularly relevant in Japan, where the rapidly aging population is expected to face a significant increase in dementia cases, with predictions suggesting that approximately 15% of adults aged 65 and over will develop dementia by 2040 [1]. Recent validation studies of OF-5 in community-dwelling older adults have demonstrated its effectiveness in identifying individuals at risk of health decline [8], suggesting its potential utility in cognitive decline risk screening.

Several limitations warrant mention. First, because our design is cross-sectional, temporal ordering and causality cannot be established; prospective cohorts are required to determine whether oral frailty precedes, follows, or co-evolves with the observed brain changes. Second, participants were drawn from a single Japanese community (Nakajima, Ishikawa), so external validity to other ethnic, cultural, or socioeconomic settings is uncertain. Third, data on dental conditions such as periodontitis and denture use, as well as systemic inflammatory biomarkers (e.g., C-reactive protein) and genetic risk markers (e.g., APOE ε4 status), were not available. These factors may independently influence both oral frailty and brain structural changes and should be incorporated into future sensitivity analyses to more fully account for potential confounding pathways. Fourth, although our analysis adjusted for many confounders, selection bias is possible due to non-random recruitment and volunteer effects. Fifth, dietary intake was measured using a food frequency questionnaire (FFQ), which is subject to recall bias and may not fully capture variability in nutrient consumption. Additionally, while our findings suggest that the revised OF-5 may improve detection of dementia-related brain changes, we were unable to calculate formal diagnostic performance metrics such as the area under the curve (AUC), net reclassification improvement (NRI), or integrated discrimination improvement (IDI) due to data limitations. Future work with more complete diagnostic outcome data is needed to validate the screening utility of the revised OF-5 using these standard comparative tools.

Finally, although the revised OF-5 combines dentist-verified tooth count with participant self-report, some checklist items remain subjective; future work should confirm inter-rater reliability and explore objective digital assessments.

## 5. Conclusions

This study demonstrates that the revised Oral Frailty 5-item Checklist (OF-5), which includes a criterion for severe tooth loss (≤9 teeth), is more effective than the original version in detecting early brain structural changes associated with dementia risk in cognitively unimpaired older adults. These associations remained consistent even after accounting for nutrition and diet, indicating that oral frailty may reflect underlying neurological vulnerability.

The revised OF-5 is a simple, non-invasive screening tool that can be easily implemented in clinical and community settings. Its ability to detect preclinical brain changes highlights its potential value in early dementia risk identification and preventive care strategies. As the global population ages, tools like this can contribute meaningfully to public health efforts aimed at delaying or preventing cognitive decline.

## Figures and Tables

**Table 1:** Oral frailty checklist.

Oral Frailty Component	Assessment Method	Definition of Oral Frailty	
		OF-5 Score 2 Points or More	Revised OF-5 Score 2 Points or More
Fewer teeth	Count by dentist	≤19 = 1 point	≤9 = 1 point
Chewing difficulty	Do you have difficulty eating hard foods compared to six months ago?	Yes = 1 point	Yes = 1 point
Swallowing difficulty	Do you sometimes choke on tea, soup, etc.?	Yes = 1 point	Yes = 1 point
Dry mouth	Do you feel dry mouth?	Yes = 1 point	Yes = 1 point
Pronunciation difficulty	Do you have trouble pronouncing words clearly in everyday conversation?	Yes = 1 point	Yes = 1 point

**Table 2:** Participant characteristics according to oral frailty status. SD, standard deviation; LDL-chol, low-density lipoprotein cholesterol; HDL-chol, high-density lipoprotein cholesterol, BMI; body mass index. * *p* < 0.05 versus the robust group.

Variables	Total	OF-5		Revised OF-5	
		Robust	Oral Frail	Robust	Oral Frail
N	732	394	338	467	265
Age, mean (SD), y	70.32 (6.54)	69.08 (5.80)	71.75 (6.97) *	69.22 (5.82)	72.25 (7.18) *
Men, n (%)	315 (43.0)	173 (43.9)	142 (42.0)	195 (41.8)	120 (45.3)
Women, n (%)	417 (56.9)	221 (56.1)	196 (58)	272 (58.2)	145 (54.7)
Hypertension, n (%)	384 (52.4)	204 (51.8)	180 (53.3)	244 (52.2)	140 (52.8)
Serum LDL-chol, mean (SD), mg/dL	115.67 (32.49)	116.71 (32.36)	114.50 (32.70)	116.94 (33.15)	113.47 (31.25)
Serum HDL-chol, mean (SD), mg/dL	61.05 (15.17)	62.46 (16.08)	59.50 (13.80) *	62.08 (15.65)	59.36 (14.05) *
Diabetes mellitus, n (%)	113 (15.4)	57 (14.5)	56 (16.6)	65 (13.9)	48 (18.1)
Formal education, mean (SD), y	11.34 (2.24)	11.39 (2.17)	11.31 (2.31)	11.41 (2.16)	11.25 (2.38)
BMI, mean (SD)	23.85 (3.39)	23.87 (3.23)	23.81 (3.43)	23.80 (3.22)	23.86 (3.50)
Smoking, n (%)	81 (11.1)	36 (9.1)	45 (13.3)	47 (10.1)	34 (12.8)
Exercise habits, n (%)	322 (43.9)	181 (46.1)	141 (42.1)	218 (46.9)	104 (39.5) *
Oral frailty component					
Fewer teeth ≤ 19, n (%)	397 (54.2)	109 (27.7)	288 (85.2) *	182 (39)	215 (81.1) *
Fewer teeth ≤ 9, n (%)	240 (32.7)	58 (14.7)	182 (53.8) *	58 (12.4)	182 (68.7) *
Chewing difficulty, n (%)	369 (50.3)	74 (18.8)	295 (87.3) *	134 (28.7)	235 (88.7) *
Swallowing difficulty, n (%)	200 (27.3)	44 (11.2)	156 (46.2) *	53 (11.3)	147 (55.5) *
Dry mouth, n (%)	82 (11.2)	9 (2.3)	73 (21.6) *	12 (2.6)	70 (26.4) *
Pronunciation difficulty, n (%)	2 (0.3)	0 (0)	2 (0.6)	1 (0.2)	1 (0.4)

**Table 3:** Association between oral frailty based on the OF-5 or revised OF-5 scores and regional brain volume in cognitively unimpaired individuals. Note. Values are presented as mean values (95% confidence interval). In the multivariable-adjusted model, the values were adjusted for age, sex, educational level, hypertension, diabetes mellitus, BMI, smoking, exercise habits, and LDL and HDL cholesterol levels. * FWE-corrected *p* < 0.05 versus the robust group. eTIV; estimated total intracranial volume; TBV, total brain volume; WMH, white matter hyperintensity.

Variables	OF-5			Revised OF-5		
	Robust	Oral Frail	−Log10 (*p*-Value)	Robust	Oral Frail	−Log10 (*p*-Value)
Brain volume to eTIV, %						
TBV	58.99 (58.69–59.29)	58.81 (58.47–59.14)	0.36	59.06 (58.78–59.34)	58.63 (58.25–59.01)	1.12
WMH	0.309 (0.278–0.339)	0.344 (0.310–0.377)	0.87	0.305 (0.277–0.333)	0.360 (0.322–0.398) *	**1.63**
Frontal lobe	9.127 (9.061–9.193)	9.103 (9.030–9.176)	0.2	9.133 (9.072–9.194)	9.085 (9.002–9.168)	0.44
Pars triangularis gyrus	0.411 (0.405–0.416)	0.405 (0.399–0.411)	0.78	0.412 (0.407–0.416)	0.402 (0.395–0.409) *	**1.56**
Temporal lobe	6.010 (5.964–6.055)	5.981 (5.930–6.031)	0.38	6.012 (5.970–6.054)	5.968 (5.911–6.025)	0.65
Temporal lobe, medial	1.855 (1.839–1.872)	1.832 (1.814–1.851)	1.13	1.856 (1.841–1.871)	1.824 (1.803–1.845) *	**1.83**
Fusiform gyrus	1.109 (1.097–1.121)	1.088 (1.075–1.101) *	**1.69**	1.111 (1.100–1.121)	1.080 (1.065–1.095) *	**2.86**
Temporal lobe, lateral	4.154 (4.120–4.189)	4.148 (4.110–4.187)	0.08	4.156 (4.124–4.188)	4.144 (4.101–4.187)	0.18
Parietal lobe	5.999 (5.954–6.044)	6.013 (5.964–6.062)	0.17	6.008 (5.967–6.049)	6.000 (5.944–6.056)	0.09
Occipital lobe	2.428 (2.407–2.450)	2.428 (2.404–2.452)	0.01	2.432 (2.412–2.452)	2.422 (2.395–2.448)	0.26
Insula	0.829 (0.821–0.836)	0.832 (0.824–0.841)	0.24	0.829 (0.822–0.836)	0.833 (0.823–0.842)	0.27
Cingulate cortex	1.135 (1.123–1.147)	1.125 (1.111–1.138)	0.57	1.134 (1.123–1.145)	1.122 (1.107–1.137)	0.68
Hippocampus	0.482 (0.476–0.487)	0.483 (0.477–0.489)	0.16	0.481 (0.476–0.486)	0.485 (0.478–0.492)	0.36
Amygdala	0.165 (0.163–0.167)	0.165 (0.162–0.167)	0.06	0.166 (0.164–0.167)	0.164 (0.161–0.167)	0.48

**Table 4:** Association between oral frailty based on the OF-5 or revised OF-5 scores and nutrient intake in cognitively unimpaired individuals. Note. Values are presented as mean values (95% confidence interval). In the multivariable-adjusted model, the values were adjusted for age, sex, educational level, hypertension, diabetes mellitus, BMI, smoking, exercise habits, and LDL and HDL cholesterol levels. eTIV; estimated total intracranial volume; TBV, total brain volume; WMH, white matter hyperintensity. Omitted in the figure are nutrients intake with −log10 (*p* value) < 1.301.

Variables	OF-5		
	Robust 7	Oral Frail	−Log10 (*p*-Value)
Total dietary fiber, g	13.86 (13.36–14.35)	12.46 (11.91–13.01)	3.54
Insoluble dietary fiber, g	9.47 (9.13–9.82)	8.57 (8.19–8.94)	3.22
Surplus ammonia, mg	19.32 (18.33–20.30)	16.72 (15.63–17.81)	3.15
Iodine, µg	2250.91 (2098.87–2402.95)	1858.06 (1690.46–2025.67)	3.07
Nitrate ion, g	0.23 (0.21–0.24)	0.20 (0.18–0.21)	3.03
Soluble dietary fiber, g	3.14 (3.01–3.26)	2.84 (2.71–2.98)	2.65
Potassium, mg	2886.11 (2793.24–2978.98)	2668.51 (2566.14–2770.89)	2.61
Vitamin K, µg	286.75 (273.58–299.92)	256.36 (241.84–270.88)	2.54
Water: fatty acids, g	1109.14 (1069.12–1149.16)	1021.63 (977.51–1065.75)	2.33
Folic acid, µg	373.26 (358.12–388.39)	341.05 (324.36–357.74)	2.23
Vitamin c, mg	113.88 (108.70–119.06)	103.03 (97.33–108.74)	2.17
Magnesium, mg	382.90 (370.15–395.65)	356.80 (342.74–370.85)	2.09
Beta-carotene equivalent, µg	3849.57 (3639.63–4059.50)	3419.49 (3188.06–3650.91)	2.09
Beta-carotene, µg	3125.23 (2945.71–3304.74)	2763.25 (2565.36–2961.14)	2.04
Water: carbon hydrate, g	1007.86 (970.05–1045.66)	933.38 (891.70–975.05)	1.97
Copper, mg	1.19 (1.16–1.22)	1.13 (1.09–1.16)	1.91
Unidentified fatty acid, mg	40.74 (37.83–43.66)	35.17 (31.96–38.38)	1.88
Alpha-carotene, µg	623.38 (585.53–661.23)	552.45 (510.73–594.17)	1.82
18:4 n-3 octadecatetraenoic acid, mg	95.72 (88.91–102.54)	84.46 (76.94–91.97)	1.49
Niacin, mg	18.57 (17.86–19.28)	17.40 (16.61–18.19)	1.47
Variables	Revised OF-5		
	Robust	Oral frail	−Log10 (*p*-value)
Iodine, µg	2221.82 (2082.24–2361.40)	1802.26 (1612.03–1992.49)	3.2
Total dietary fiber, g	13.70 (13.25–14.16)	12.35 (11.73–12.97)	3.13
Insoluble dietary fiber, g	9.36 (9.04–9.67)	8.53 (8.10–8.96)	2.57
Vitamin K, µg	283.95 (271.85–296.05)	253.04 (236.55–269.53)	2.44
Folic acid, µg	371.16 (357.26–385.06)	335.95 (317.01–354.88)	2.41
Soluble dietary fiber, g	3.10 (2.99–3.22)	2.83 (2.67–2.98)	2.27
Surplus ammonia, mg	18.93 (18.02–19.84)	16.71 (15.47–17.95)	2.27
Potassium, mg	2858.63 (2773.21–2944.05)	2658.27 (2541.86–2774.68)	2.12
Nitrate ion, g	0.22 (0.21–0.23)	0.20 (0.18–0.21)	2.12
Copper, mg	1.19 (1.16–1.22)	1.12 (1.07–1.16)	2.11
Manganese, mg	3.70 (3.58–3.83)	3.42 (3.25–3.59)	2.1
Iron, mg	8.45 (8.20–8.69)	7.91 (7.58–8.25)	1.87
Vitamin c, mg	112.60 (107.84–117.36)	102.35 (95.86–108.84)	1.85
Magnesium, mg	379.96 (368.25–391.68)	354.91 (338.94–370.88)	1.83
Molybdenum, µg	233.62 (227.32–239.92)	220.59 (212.00–229.18)	1.73
Water, g	1505.45 (1453.53–1557.38)	1405.44 (1334.68–1476.21)	1.55
18:1 count, mg	15,856.74 (15,324.46–16,389.02)	16,852.69 (16,127.28–17,578.09)	1.48
Beta-carotene, µg	3067.39 (2902.22–3232.56)	2768.26 (2543.16–2993.36)	1.41
Beta-carotene equivalent, µg	3774.38 (3581.15–3967.62)	3437.19 (3173.85–3700.54)	1.33

**Table 5:** Association between oral frailty based on the OF-5 or revised OF-5 scores and food and beverage intake in cognitively unimpaired individuals. Note. Values are presented as mean values (95% confidence interval). In the multivariable-adjusted model, the values were adjusted for age, sex, educational level, hypertension, diabetes mellitus, BMI, smoking, exercise habits, and LDL and HDL cholesterol levels. eTIV; estimated total intracranial volume; TBV, total brain volume; WMH, white matter hyperintensity. Omitted in the figure are nutrients intake with −log10 (*p* value) < 1.301.

Variables	OF-5		
	Robust	Oral Frail	−Log10 (*p*-Value)
Other vegetables, g	153.37 (144.89–161.85)	130.45 (121.10–139.80)	3.32
Vegetables not high in beta-carotene, g	153.08 (144.64–161.53)	130.47 (121.16–139.78)	3.26
Seaweed, g	14.82 (13.80–15.84)	12.17 (11.04–13.29)	3.1
Nuts and bolts, g	3.12 (2.60–3.65)	1.76 (1.18–2.34)	3.1
Lard, g	6.09 (5.56–6.61)	7.40 (6.83–7.98)	2.94
Cider, g	3.51 (−0.39–7.40)	12.24 (7.95–16.54)	2.43
Fats and oils, g	7.81 (7.20–8.42)	9.11 (8.44–9.78)	2.25
Rolled oats, g	0.57 (0.35–0.79)	0.12 (−0.12–0.36)	2.13
Cheese, g	3.86 (3.36–4.37)	2.95 (2.39–3.51)	1.72
Udon noodles, g	9.14 (6.12–12.17)	14.54 (11.21–17.88)	1.68
Vegetables high in beta-carotene, g	72.37 (67.78–76.95)	64.29 (59.23–69.34)	1.65
Green fish, g	33.33 (30.55–36.12)	28.44 (25.37–31.51)	1.64
Japanese dressing type seasoning, g	1.33 (0.99–1.67)	0.75 (0.38–1.13)	1.57
White sugar, g	7.80 (7.42–8.18)	8.45 (8.02–8.87)	1.53
Noodles, g	45.45 (39.88–51.03)	54.85 (48.70–60.99)	1.53
Margarine, g	0.70 (0.48–0.92)	1.05 (0.81–1.29)	1.4
Yogurt nonfat-sweetened, g	12.42 (9.37–15.47)	17.16 (13.80–20.52)	1.36
Soybeans natto, g	15.90 (14.50–17.31)	13.72 (12.17–15.27)	1.35
Potato, g	24.15 (22.63–25.67)	21.81 (20.14–23.49)	1.33
All kinds of potatoes, g	24.15 (22.63–25.67)	21.81 (20.14–23.49)	1.33
Variables	Revised OF-5		
	Robust	Oral frail	−Log10 (*p*-value)
Seaweed, g	14.61 (13.68–15.55)	11.81 (10.53–13.09)	3.17
Fats and oils, g	7.84 (7.28–8.39)	9.42 (8.66–10.17)	2.9
Rolled oats, g	0.56 (0.36–0.76)	0.01 (−0.26–0.28)	2.73
Nuts and bolts, g	2.96 (2.48–3.44)	1.68 (1.03–2.34)	2.58
Other vegetables, g	149.96 (142.15–157.77)	130.30 (119.66–140.95)	2.38
Vegetables not high in beta-carotene, g	149.70 (141.92–157.48)	130.36 (119.76–140.96)	2.34
Blended oil, g	6.80 (6.33–7.27)	7.86 (7.22–8.50)	2.02
Soybeans natto, g	15.93 (14.64–17.23)	13.06 (11.30–14.82)	1.95
Lard, g	6.30 (5.82–6.79)	7.37 (6.72–8.03)	1.94
Canned coffee, g	14.42 (6.68–22.15)	30.90 (20.36–41.45)	1.82
Potato, g	24.12 (22.72–25.51)	21.23 (19.33–23.13)	1.74
All kinds of potatoes, g	24.12 (22.72–25.51)	21.23 (19.33–23.13)	1.74
Udon noodles, g	9.71 (6.93–12.49)	15.01 (11.22–18.80)	1.52
Green fish, g	32.75 (30.19–35.31)	28.14 (24.66–31.63)	1.4
Cider, g	5.18 (1.59–8.77)	11.61 (6.72–16.50)	1.39
French dressing, g	1.76 (1.47–2.04)	2.26 (1.87–2.65)	1.34
Instant noodles, g	1.63 (0.75–2.50)	3.14 (1.94–4.34)	1.31

**Table 6:** Association between oral frailty based on the OF-5 or revised OF-5 scores and regional brain volume in cognitively unimpaired individuals, with additional adjustment for nutrient intake. Note. Values are presented as mean values (95% confidence interval). In the multivariable-adjusted model, the values were adjusted for age, sex, educational level, hypertension, diabetes mellitus, BMI, smoking, exercise habits, and LDL and HDL cholesterol levels, and nutrient intake variables with −log10 (*p*-value) > 1.301 in Table 4. * FWE-corrected *p* < 0.05 versus the robust group. eTIV; estimated total intracranial volume; TBV, total brain volume; WMH, white matter hyperintensity.

Variables	OF-5			Revised OF-5		
	Robust	Oral Frail	−Log10 (*p*-Value)	Robust	Oral Frail	−Log10 (*p*-Value)
Brain volume to eTIV, %						
TBV	58.95 (58.64–59.26)	58.85 (58.51–59.19)	0.17	59.03 (58.75–59.31)	58.68 (58.30–59.07)	0.79
WMH	0.304 (0.273–0.335)	0.349 (0.315–0.384)	1.22	0.302 (0.273–0.330)	0.366 (0.327–0.405) *	**1.96**
Frontal lobe	9.126 (9.059–9.193)	9.103 (9.029–9.177)	0.18	9.133 (9.072–9.195)	9.084 (9.000–9.168)	0.44
Pars triangularis gyrus	0.411 (0.405–0.416)	0.405 (0.399–0.411)	0.76	0.412 (0.407–0.417)	0.401 (0.395–0.408) *	**1.78**
Temporal lobe, medial	1.854 (1.837–1.871)	1.833 (1.815–1.852)	0.96	1.856 (1.841–1.872)	1.824 (1.803–1.845) *	**1.78**
Fusiform gyrus	1.109 (1.097–1.121)	1.088 (1.075–1.102) *	**1.53**	1.111 (1.100–1.122)	1.080 (1.064–1.095) *	**2.8**
Temporal lobe, lateral	4.152 (4.117–4.187)	4.152 (4.113–4.190)	0	4.155 (4.123–4.187)	4.146 (4.103–4.190)	0.11
Parietal lobe	5.998 (5.952–6.043)	6.015 (5.964–6.065)	0.2	6.007 (5.966–6.049)	6.002 (5.944–6.059)	0.06
Occipital lobe	2.427 (2.406–2.449)	2.429 (2.405–2.453)	0.03	2.431 (2.411–2.451)	2.423 (2.395–2.450)	0.2
Insula	0.829 (0.822–0.837)	0.832 (0.823–0.840)	0.19	0.830 (0.823–0.837)	0.831 (0.822–0.841)	0.09
Cingulate cortex	1.135 (1.123–1.147)	1.124 (1.111–1.138)	0.55	1.135 (1.124–1.146)	1.122 (1.106–1.137)	0.75
Hippocampus	0.481 (0.476–0.487)	0.484 (0.478–0.490)	0.36	0.481 (0.476–0.486)	0.486 (0.479–0.493)	0.59
Amygdala	0.165 (0.163–0.167)	0.165 (0.163–0.167)	0.03	0.165 (0.163–0.167)	0.164 (0.161–0.167)	0.39

**Table 7:** Association between oral frailty based on the OF-5 or revised OF-5 scores and regional brain volume in cognitively unimpaired individuals, with additional adjustment for food and beverage intake. Note. Values are presented as mean values (95% confidence interval). In the multivariable-adjusted model, the values were adjusted for age, sex, educational level, hypertension, diabetes mellitus, BMI, smoking, exercise habits, and LDL and HDL cholesterol levels, and food and beverage intake variables with −log10 (*p*-value) > 1.301 in Table 5. * FWE-corrected *p* < 0.05 versus the robust group. eTIV; estimated total intracranial volume; TBV, total brain volume; WMH, white matter hyperintensity.

Variables	OF-5			Revised OF-5		
	Robust	Oral Frail	−Log10 (*p*-Value)	Robust	Oral Frail	−Log10 (*p*-Value)
Brain volume to eTiv, %						
TBV	58.94 (58.63–59.26)	58.86 (58.52–59.20)	0.14	59.01 (58.73–59.29)	58.72 (58.33–59.11)	0.59
WMH	0.309 (0.277–0.340)	0.344 (0.309–0.378)	0.8	0.304 (0.276–0.333)	0.361 (0.322–0.401) *	**1.57**
Frontal lobe	9.124 (9.056–9.192)	9.106 (9.031–9.181)	0.13	9.131 (9.069–9.193)	9.088 (9.002–9.174)	0.36
Pars triangularis gyrus	0.411 (0.406–0.417)	0.405 (0.399–0.411)	0.87	0.412 (0.407–0.417)	0.401 (0.394–0.408) *	**1.93**
Temporal lobe	6.005 (5.958–6.052)	5.986 (5.934–6.038)	0.22	6.008 (5.965–6.050)	5.976 (5.917–6.035)	0.39
Temporal lobe, medial	1.855 (1.838–1.872)	1.832 (1.813–1.851)	1.07	1.855 (1.839–1.870)	1.827 (1.806–1.848) *	**1.32**
Fusiform gyrus	1.109 (1.097–1.122)	1.088 (1.074–1.101) *	**1.6**	1.110 (1.099–1.121)	1.081 (1.066–1.097) *	**2.32**
Temporal lobe, lateral	4.150 (4.115–4.185)	4.154 (4.115–4.193)	0.05	4.153 (4.121–4.185)	4.149 (4.105–4.194)	0.05
Parietal lobe	5.994 (5.948–6.040)	6.019 (5.968–6.070)	0.31	6.004 (5.963–6.046)	6.007 (5.949–6.065)	0.02
Occipital lobe	2.426 (2.404–2.448)	2.430 (2.406–2.455)	0.08	2.431 (2.411–2.451)	2.423 (2.395–2.450)	0.19
Insula	0.829 (0.821–0.837)	0.832 (0.823–0.840)	0.18	0.830 (0.823–0.837)	0.832 (0.822–0.842)	0.15
Cingulate cortex	1.135 (1.123–1.147)	1.124 (1.111–1.138)	0.56	1.134 (1.123–1.145)	1.122 (1.107–1.138)	0.63
Hippocampus	0.481 (0.476–0.487)	0.484 (0.478–0.490)	0.25	0.480 (0.475–0.486)	0.487 (0.480–0.494)	0.74
Amygdala	0.165 (0.163–0.167)	0.165 (0.162–0.167)	0.03	0.165 (0.163–0.167)	0.164 (0.161–0.167)	0.31

## Data Availability

Because of the inclusion of sensitive medical information that could compromise participant privacy, the data analyzed in this study cannot be made publicly available. However, anonymized data can be accessed upon reasonable request and with permission from the corresponding author.
